# A current and future perspective on T cell receptor repertoire profiling

**DOI:** 10.3389/fgene.2023.1159109

**Published:** 2023-06-20

**Authors:** Yiran Shen, Alexandria Voigt, Xuebing Leng, Amy A. Rodriguez, Cuong Q. Nguyen

**Affiliations:** ^1^Department of Infectious Diseases and Immunology, College of Veterinary Medicine, University of Florida, Gainesville, FL, United States; ^2^Department of Microbiology and Immunology, Miller School of Medicine, University of Miami, Miami, FL, United States; ^3^ Department of Oral Biology, College of Dentistry, University of Florida, Gainesville, FL, United States; ^4^ Center of Orphaned Autoimmune Diseases, University of Florida, Gainesville, FL, United States

**Keywords:** TCR, RNA, autoimmune, HLA, sequencing

## Abstract

T cell receptors (TCR) play a vital role in the immune system’s ability to recognize and respond to foreign antigens, relying on the highly polymorphic rearrangement of TCR genes. The recognition of autologous peptides by adaptive immunity may lead to the development and progression of autoimmune diseases. Understanding the specific TCR involved in this process can provide insights into the autoimmune process. RNA-seq (RNA sequencing) is a valuable tool for studying TCR repertoires by providing a comprehensive and quantitative analysis of the RNA transcripts. With the development of RNA technology, transcriptomic data must provide valuable information to model and predict TCR and antigen interaction and, more importantly, identify or predict neoantigens. This review provides an overview of the application and development of bulk RNA-seq and single-cell (SC) RNA-seq to examine the TCR repertoires. Furthermore, discussed here are bioinformatic tools that can be applied to study the structural biology of peptide/TCR/MHC (major histocompatibility complex) and predict antigenic epitopes using advanced artificial intelligence tools.

## 1 Introduction

T cell function is initiated by recognition of a peptide antigen in a specific interaction via the T cell receptor (TCR) in the context of the major histocompatibility complex (MHC) expressed on antigen-presenting cells (APC). TCRs are heterodimeric membrane proteins that are composed of two chains, αβ or γδ. The *α* chain is made up of the variable (V), joining (J) and constant (C) segments, and the *β* chain contains the V, D (diversity), J, and C segments. The gene segment organization of the TCRγ and TCRδ chains is similar to that of the αβ TCR. TCR development in the thymus is critical for development of a functional immune system. The gene rearrangement of a TCR involves the selection of immature T cells in the thymus maturing to become functional T cells that recognize foreign molecules and respond to them appropriately. The mature T cells undergo positive and negative selection, in which they are presented with self-antigens from the thymus for affinity selection to prevent autoreactive TCR repertoires. This process helps to ensure that only mature T cells respond to foreign antigens exclusively are allowed to survive and develop (Schatz and Ji, 2011). The rearrangement leads to a vast diversity of TCR repertoires capable of recognizing almost any peptide presented by MHC molecules (Mitchell and Michels, 2020). The diversity of the αβ TCR from the unique pairing of various gene segments or loci generates on the order of 10^18^ or more possible combinations (Murphy and Weaver, 2022). Once naïve T cells encounter the peptide-MHC complex (pMHC) presented by an APC, these T cells will start to undergo clonal expansion while retaining the initial TCR sequence (Huang et al., 2019).

T cells and their receptors are crucial in autoimmunity. Recognition of autoantigens by T cells with self-reactive TCRs can result in tissue-specific damage of systemic autoimmune diseases (Seiringer et al., 2022). A fitting model for this process is Sjögren’s disease (SjD), which is a debilitating disease affecting as many as 3.1 million individuals in the United States(Kassan and Moutsopoulos, 2004; Helmick et al., 2008; Nguyen and Peck, 2009). In addition to secretory dysfunction resulting in dry mouth (xerostomia) and dry eyes (keratoconjunctivitis sicca), symptoms can manifest systemically to the skin, gastrointestinal tract, lungs, blood vessels, liver, pancreas, kidneys, vagina, and peripheral and central nervous system (Cornec et al., 2014; Voigt et al., 2014; Nocturne and Mariette, 2015; Voigt and Nguyen, 2015). The TCR usage of individual αβ T cells showed that the TCR-Vα repertoire of infiltrating T cells is restricted with limited heterogeneity. Specifically, Vα usage of TCR genes, including Vα17.1, Vα2, and Vα11.1, were found dominantly in salivary glands (SG) and not peripheral blood mononuclear cells (PBMCs) (Sumida et al., 1994a). A study (Joachims et al., 2016) demonstrated that glandular memory T cells showed a number of TCRs, specifically TRAV8-2, 12-3, 12-2, 16, and TRBV30, 20-1, 19, 7-6, 14, 20-1, 3-1, and 24-1. In the non-obese diabetic (NOD) mouse model, it has been shown that 15% of the TRBV gene is Vβ8.1.2, followed by Vβ6, Vβ10b, Vβ11, Vβ2, and Vβ7 (Sumida et al., 1994b; Skarstein et al., 1995). During autoimmune sialadenitis or early stages of the disease, the predominant expression of the Vβ8 gene increased over time in the MRL/lpr strain. Although the self-antigen was not identified, the usage of TCR-Vβ elements being restricted according to the stage of the disease indicates a clonal selection of antigen-specific TCR in the SG, suggesting that the diversity of TCR repertoires is disease- and stage-dependent (Hayashi et al., 1995).

The studies, as mentioned earlier, applied various techniques to study TCR and cell types based on transcriptomic data. To advance beyond the transcriptome, one must be able to decipher the antigen or autoantigens presented to the T cells, which will further our understanding of the immunological mechanism underpinning the onset and progression as well as improve clinical diagnostics and therapeutics. The overall objective of this review is to describe the latest technological advances that have had a significant impact on profiling TCR repertoires and concomitantly linking them to the cellular transcriptomic profiles of the target cells. In addition, we discuss predictive modeling based on particular antigenic epitopes and TCR repertoires.

## 2 Development of RNA sequencing (RNA-seq) technology to identify TCR repertoire

### 2.1 Single-stranded RNA-seq

**FIGURE 1:**
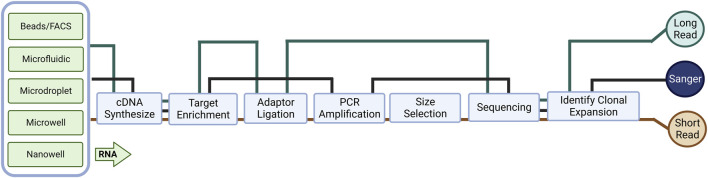
An overview of library preparation methods for different cell preparation and RNA sequencing (RNA-seq) methods. The complexity and bias of library preparation vary depending on the specific method used.

Molecular cloning and Sanger sequencing were the first methods to study immune repertoires at the nucleotide sequence level (Figure 1). Early work by Sant’Angelo et al. (1998) showed that the complementarity determining region 3 (CDR3) can be obtained by designing primers for the paired V- and C-region’s primary and restriction fragment length polymorphism (RFLP) with nested PCR amplification. They sequenced CDR3 regions, analyzed TCRα chains from different TCRβ chain-transgenic mice, and constructed a molecular map of T cell development; they identified the precise stage of positive selection that occurs early in thymocyte differentiation. Later, Correia-Neves et al. (2001) designed a mouse line by combining the TCRβ transgene with the TCRα minilocus consisting of a single V and two J gene segments. They also performed nested PCR by paired primers designed with a similar concept to determine the diversity of CDR3α. This approach allowed them to follow the fate of T cells with different TCR sequences, thus enabling them to study the selection and evolution of the T cell repertoires. A widely used method is multiplex PCR, wherein multiple primers are designed to amplify all possible V segments using degenerate primers and conserved region primers. Primer bias can occur with this approach which distorts the resulting TCR repertoires, therefore sequencing the final cDNA must be done to confirm the identify the targeted receptors (Liu et al., 2016). Unbiased 5′-Rapid Amplification of cDNA Ends (5′ RACE) is alternative method, as it amplifies TCR genes using only one primer targeting a constant region and a universal primer attached to the 5′ end (Mamedov et al., 2013). Recently, Cook et al. (2020) used 5′ RACE PCR to amplify TCRβ chain and Sanger sequencing to analyze the TCR repertoire of the regulatory CD4^+^ T cell (Treg) population and found that the TCR repertoire of gluten-specific CD39^+^ Tregs in celiac disease patients was oligoclonal compared to healthy controls, suggesting that the repertoire of gluten-specific CD39^+^ Tregs may be driven by the specific antigen and the corresponding human leukocyte antigen (HLA) restriction. Unlike multiplex PCR, which can use both genomic DNA and RNA as input, 5′-RACE can only be applied to RNA samples, and the presence of short DNA fragments in the 5′-RACE library may result in sequencing results that do not effectively present regularly recombinant TCR sequences (Lin et al., 2020).

Even though there are many options and optimizations in the methods of molecular cloning to generate sequencing materials, Sanger sequencing is limited due to low throughput and can only sequence a small number of TCRs in a given batch. In particular, during bulk analysis, PCR technologies can only amplify and sequence one strand at a time, thus losing the pairwise information of intact immune repertoires, making it impossible to determine the antigenic specificity of pathogenic TCR information subsequently. Meanwhile, most early TCR profiling studies were based on analysis of the CDR3 region; however, full-length sequencing includes other regions, e.g., CDR1 and CDR2, involves in antigen receptor binding affinity and/or downstream signaling, and allows direct cloning and expression of identified and selected receptors for further experiments (Mazzotti et al., 2022). The widespread use of next-generation sequencing (NGS) based RNA-seq has shaped our understanding of many aspects of biology. Unlike genomic DNA-based applications, RNA-seq provides comprehensive gene expression information from mRNA in addition to the immune repertoire. Short-read RNA-seq is cheaper and easier to perform than microarrays and produces comprehensive, high-quality, less biased data in a shorter time, thus allowing easy determination of clonal expansion in TCR replication. More importantly, in TCR sequencing, the choice of RNA rather than DNA as starting material avoids small sample size of genomic DNA (gDNA), as well as reducing background interference and primer bias from amplification of V and J fragments that are not involved in recombination but remained in gDNA.

In autoimmune diseases, current commercial services can identify the most frequent single-strand used TCR from patients, typically starting with multiplex PCR to amplify all Vα or Vβ regions followed by short-read RNA-seq to confirm the clonal expansion of immune cells. Muraro et al. (2014) used high-throughput deep TCRβ sequencing provided by ImmunoSEQ to assess millions of individual TCRs in multiple sclerosis (MS) patients with poor prognosis per hematopoietic stem cell transplantation (HSCT) treatment and found that the combination of high-dose immunosuppressive therapy (HDIT) and autologous HSCT had a unique and independent effect on reconstituting CD4^+^ and CD8^+^ repertoires, which supports the view that TCR repertoire diversity is critical for reestablishing immune tolerance. However, ImmunoSEQ is a gDNA-based service, which is accomplished by pre-running a synthetic immune repertoire that represents all combinations of V-J genes, before selecting and adjusting primer concentrations to reduce bias during amplification. The most widely used commercial RNA-based kit is iRepertoire, Chang et al. (2019) used iRepertoire to sequence the TCRβ CDR3 region to determine the role of T cell profiles in rheumatoid arthritis patients receiving different biologic disease-modifying antirheumatic drugs (bDMARDs). An index of clonality of the TCRβ repertoires in RA patients was found to be negatively correlated with age, while a trend toward increased disease activity was observed with reduced TCRβ repertoire diversity following bDMARDs treatment. Using the same technique, Amoriello et al. (2020) tracked peripheral T cell subsets in 15 relapsing-remitting multiple sclerosis (RRMS) patients before and after 2 years of continuous treatment with Natalizumab (NTZ) and a single course of therapy with autologous hematopoietic stem cell transplantation (AHSCT) by high-throughput TCRβ sequencing, they found that both treatments left treatment-specific multidimensional traces in patient TCRβ repertoire dynamics related to clonal amplification, clonal diversity, and repertoire structure. A comparison of iRepertoire with other commercially available kits (MiLaboratories, Takara, NEB) is shown in Table 1. Amplification can also be performed by adding adaptor sequences into TCR multiplex PCR primers, Wang et al. (2021) first used scRNA-seq to reveal a novel Graves’ orbitopathy (GO)-specific cell type, CD4^+^ cytotoxic T lymphocytes (CTL), and to understand the clonal expansion of this CD4^+^ CTL population, they performed TCRβ CDR3 sequencing, revealing the significant clonal expansion of CD4^+^ KLRG1^+^ CTL from GO patients.

**TABLE 1:** Major commercially available kits for TCR profiling.

	Milaboratories	NEBNext^®^ immune sequencing	SMARTer TCR a/b profiling	iRepertoire
Species	Mouse, human, and monkey	Mouse and human	Mouse and human	Mouse and human
Protocol	Multiplex PCR	5′ RACE	SMART technology	arm-PCR
UMI	Yes	Yes	No	No
Input material	Up to 500 ng	10 ng–1 µg RNA or RNA- contained cells	10 ng–3 µg of RNA or 50–10,000 cells	50 ng–1 µg RNA
Sequencing	Illumina	Illumina Miseq^®^	Illumina Miseq^®^	Illumina
Analysis	MIXCR and MIGEC	Presto (Galaxy)	Any softwares	iRepertoire

SMART, Switching Mechanism at 5′ End of RNA template; arm-PCR, amplicon rescued multiplex PCR.

It is possible to sequence large cell populations in this manner, but the fact that it can only be based on single-strand RNA-seq is likewise a limiting point. Due to the presence of the D loci, the TCRβ chain has a higher combinatorial potential than the TCRα chain. Also, due to allelic exclusion (Khor and Sleckman, 2002) and the possibility of two *α* chains being expressed by the same cell (Padovan et al., 1993), the single *β* chain expressed per αβ T cell has become the main target for single-strand sequencing studies, but this introduces a sample bias.

### 2.2 Paired-stranded sequencing based on short-read single-cell RNA-seq (scRNA-seq)

Developments in wet lab technology and computing drive the adaptation and evolution of RNA-seq. In this context, single-cell-based experimental techniques can overcome the limitations of single-strand sequencing in TCRs (Hou et al., 2016). Paired TCR αβ or γδ sequences can provide additional information on p (peptide) MHC binding specificity, which is essential for the study of autoimmune disease etiology and progression. Low-put through scRNA-seq involves manually sorting and isolating individual cells by magnetic bead sorting or fluorescence-activated cell sorting (FACS) into multi-well plates. Switching Mechanism at the end of the 5′-end of the RNA Transcript (SMART)-seq (Goetz and Trimarchi, 2012), Smart-seq2 (Picelli et al., 2013), MATQ-seq (Sheng et al., 2017), CEL-seq (Hashimshony et al., 2012) and other protocols can rely on FACS sorting. After first strand cDNA synthesis, unlike non-linear PCR, platforms, for example, CEL-seq utilize *in vitro* transcription (IVT) technology, it requires an additional round of reverse transcription of the amplified RNA which results in a 3′- bias.

To process a large number of single cells simultaneously, several commercial services have introduced either microfluidic (Fluidigm C1), microdroplet (10X Genomics), microwell (Clontech, BD Rhapsody), or nanowell (ICELL8)-based platforms that allow for automated isolation, lysis, and cDNA synthesis for each cell (Figure 1). These automated platforms rely on in-house developed instrumentation, which reduces the batch effect of samples but increases costs. These platforms utilize a variety of different cell isolation techniques while differing in cell lysis, reverse transcription, amplification, transcript coverage, strand specificity, or UMI (Unique Molecular Identifier) availability (Table 2). To estimate technical differences between cells, correct the errors, and normalize data, the use of UMIs can offset differences in mRNA amplification efficiency, which can detect and quantify molecular labels of unique transcripts. Another option is the use of external RNA control consortium (ERCC) introduced into the samples to calibrate measurements and account for technical variation, which was applied in SMART-seq2 protocol but is not compatible with droplet-based platforms (Svensson et al., 2017; Baran-Gale et al., 2018).

**TABLE 2:** Current automate platform for single cell processing.

Platform	Compatible protocol	Transcript coverage	UMI
Fluidigm C1	SMART-Seq	Full-length	No
10X Genomics	Chromium	5′-/3′-	Yes
Clontech	SMART-Seq	Full-length	No
ICELL8	SMART-Seq	Full-length	No
BD Rhapsody	Whole transcriptome analysis (WTA)	3′-	Yes

**FIGURE 2:**
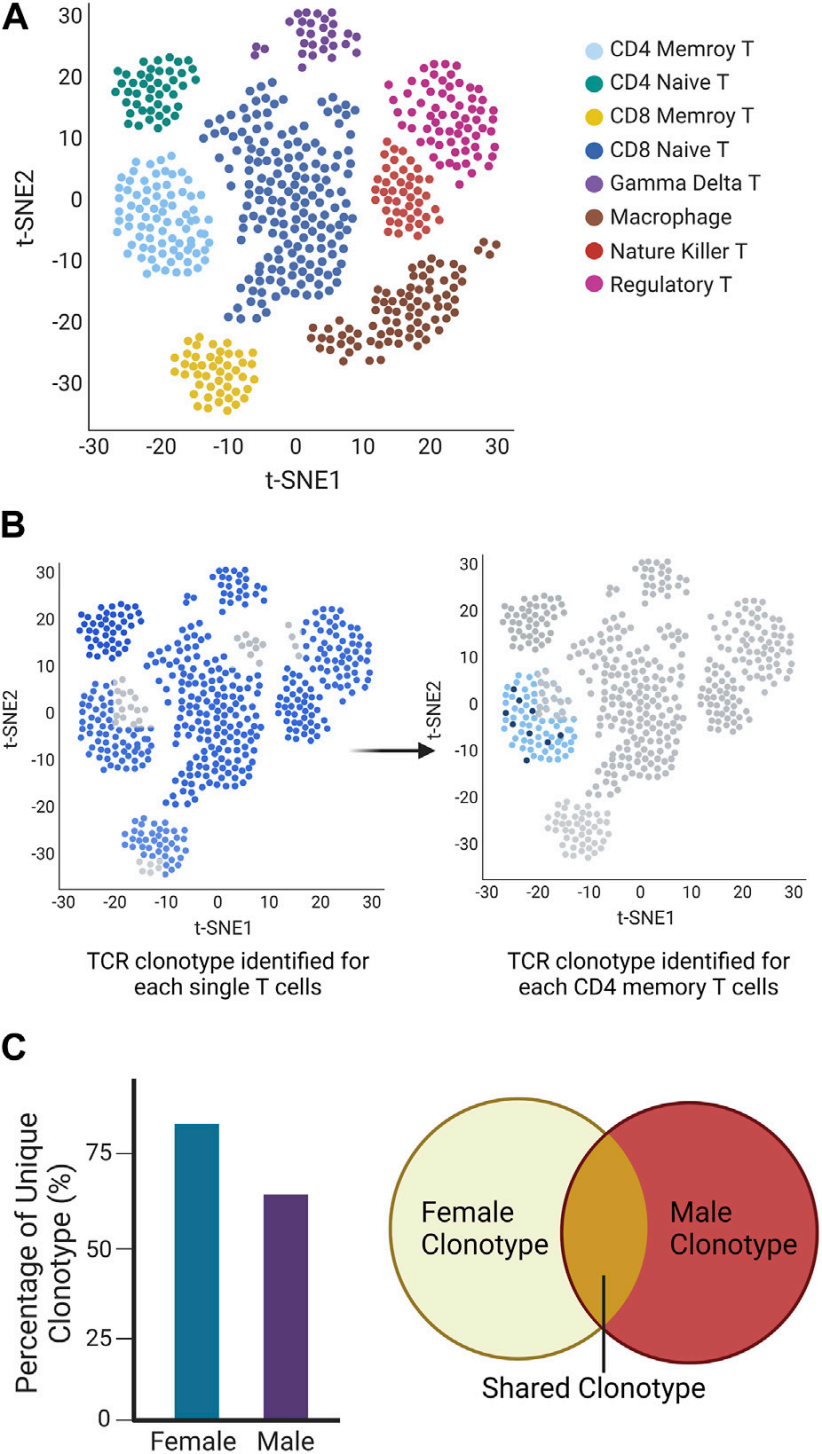
Mockup Chromium Single Cell Immune Profiling (10X Genomics) to identify the specific immune cell subsets and the expressed TCR repertoire of single T cells. **(A)** UMAP of the different cell types is classified based on different colors. **(B)** TCR repertoires in each T cell (left) and selected memory T cell (right). Each cell identified with a TCR clonotype was labeled blue, otherwise was grey (no identified TCRα/β or TCRγ/δ present). The same clonotype is marked in the same dark blue color (right). **(C)** The different clonotypes expansion between sexes showed as a percentage of unique clonotypes, and share clonotypes were presented in Venn diagram.

Still, automatic single-cell processing reduces intracellular RNA degradation and library preparation time, and scTCR-seq facilitates the exploration of the immune repertoire with great diversity. These factors together allow us to further explore key cell subpopulations and differentiation states through transcriptome analysis and to infer cell developmental trajectories at the single-cell level while providing additional information related to the TCR repertoire. We recently utilized Chromium Single Cell Immune Profiling (10X Genomics) to identify the specific immune cell subsets and the expressed TCR repertoire of single T cells. The technology combines single-cell sequencing and molecular barcoding to measure the TCR sequences expressed by individual T cells, allowing us to make detailed inferences about the composition and diversity of the immune system. In here we present a mockup figure to illustrate the whole workflow (Figure 2), we found different populations of immune cells present in the salivary glands of SjD-susceptible mice (Figure 2A). When we examined the TCR repertoire expressed by the effector CD4^+^ T cells, we were able to identify the dominant receptors (Figure 2B). We further demonstrated that males and females of the same SjD background exhibited different TCR repertoires (Figure 2C).

The use of scRNA-seq for identifying autoimmune disease-related immune repertoires has only recently emerged, including type 1 diabetes (T1D) (Linsley et al., 2021; Kasmani et al., 2022), autoimmune hepatitis (AIH) (Renand et al., 2020), primary SjD (pSjD) (Hong et al., 2020; Hou et al., 2022), and systemic lupus erythematosus (SLE) (Smita et al., 2022) (Table 3). Not only is it a direct study of the disease itself, but scTCR-seq has also been used to study T cell populations and/or related mechanisms closely associated with autoimmune disorders, allowing us to visualize the immune repertoire expressed by several cell subpopulations. In spondyloarthritis (SpA) patients, arthritogenic peptides are presented by the risk allele HLA-B*27 to antigen-specific CD8^+^ T cells to initiate or maintain an autoimmune response, Deschler et al. (2022) used scTCR-seq to analyze CD8^+^ T cells in the patient’s synovial fluid (SF) and revealed a preferential expansion of the TCR TRAV- and TRBV- families, common motifs in the CDR3 loop and identical TCR chains across patients. Follicular helper T cells are central regulators of germinal centers and contribute to the formation of pathogenic autoantibodies, Akama-Garren et al. (2021) performed scRNA-seq and scTCR-seq cells of follicular helper T cells in a mouse model of autoantibody-mediated disease, they found that a few TCR clonotypes were preferentially shared among autoimmune follicular helper T cells and the amplification correlated with differential genetic signatures in autoimmune disease. These studies have yielded paired TCR information that complements and confirms previous studies, combining transcriptome analysis with corresponding single cells provides a comprehensive definition of the immune cell population that can provide a more accurate basis for downstream functional experiments. In the recent COVID-19 outbreak, we also observed the link of autoimmune phenotypes to SARS-CoV-2 infection in children using of scTCR-seq. Multisystemic inflammatory syndrome in children (MIS-C) is a life-threatening post-infection complication that occurs unpredictably weeks after mild or asymptomatic SARS-CoV-2 infection. Patients with clinically severe MIS-C exhibit a skewed memory T cell TCR repertoire and endothelial-reactive IgG autoantibodies. Using scRNA-seq, Ramaswamy et al. (2021) analyzed PBMC from patients and found that CD4^+^ and CD8^+^ memory T cells expressing TRBV11-2 were amplified in severe MIS-C.

**TABLE 3:** scRNA-seq for identifying autoimmune disease-related TCR.

Disease	Finding	References
T1D	Islet-specific glucose-6-phosphatase catalytic subunit-related protein (IGRP)_206–214_-reactive CD8^+^ T (self-reactive T cells) in T1D may inherently have a restricted TCR library as well as a substantial TCR motif overlap	Kasmani et al. (2022)
T1D	It has identified a class of autoreactive TCRs from human IAR (islet antigen reactive) CD4^+^ T cells in patients with T1D that share the feature of germline alpha chains	Linsley et al. (2021)
AIH	Identified the central memory CD45RA^−^CD27^+^PD-1^+^CXCR5^−^CCR6^−^ CD4^+^ T cell population as the significant self-reactive CD4^+^ T cell pool in AIH	Renand et al. (2020)
pSjD	T cell receptor alpha and beta chain variable genes of TRAV13-2 and TRBV7-9 were significantly expanded in patients with pSjD	Hong et al. (2020)
pSjD	The degree of TCR clonal expansion did not differ significantly between the pSjD patients and healthy individuals. Still, the frequencies of T cells with dual TCR β-chain expression were reduced considerably in pSjD patients	Hou et al. (2022)
SLE	Found CD8^+^ kidney-infiltrating T cells (KIT) first existed in a transitional state, then clonally expanded and evolved to depletion in the kidney	Smita et al. (2022)

T1D, Type 1 diabetes; AIH, Autoimmune hepatitis; pSjD, Primary Sjögren’s disease; SLE, Systemic lupus erythematosus.

The read length of RNA-seq is much shorter than that of first-generation sequencing (e.g., Sanger sequencing), and scRNA-seq data often contain many missing values or dropouts due to the failure to amplify the original RNA input, this frequency depends mainly on the protocol. Thus, it is crucial to use appropriate methods to overcome this problem when analyzing samples. Even so, the current scRNA-seq technology allows combining RNA-seq of the same cells with paired TCR-seq, and the great advantage of simultaneously processing cell numbers is essential for identifying the characteristics of rare T cell populations. These studies have generated new insights into disease biology and demonstrated the potential of scTCR-seq for clinical applications. Furthermore, in autoimmune diseases, identifying auto-reactive T cells by scTCR-seq may provide an indirect method to identify autoantigens.

### 2.3 TCR-pMHC sequencing potential based on long-read scRNA-seq

Long-read sequencing platforms, namely, long-read cDNA and long-read RNA sequencing, can capture many full-length transcripts (1–50 kb), unlike short-read sequencing, which requires fragmentation and amplification as well as introduced the previously discussed bias; additionally, assembly with bioinformatic tools relies on an existing genomic database (Salzberg and Yorke, 2005) (Figure 1), the computational approach for *de novo* transcriptome analysis utilized by long-read sequencing is easier and more unbiased (Stark et al., 2019) Processing the whole sample eliminates the amplification bias and has the ability to detect large insertions/deletions and duplicate regions. The two most widely used commercial technologies are Pacific Biosciences’ single molecule real-time (SMRT) sequencing (average read length of HiFi reads ∼20 kb, accuracy >99.9%) and Oxford Nanopore Technologies’ nanopore sequencing (average read length of ultra-long reads ∼100 kb, accuracy of R10.4 ∼99%) (Method of the Year 2022: long-read sequencing., 2023). Specifically, no reverse transcription step is required in long-read RNA-seq, the PCR-free library-building protocol avoids guanine-cytosine (GC) bias and makes long-read sequencing platforms well-suited for studies of immune repertoires, as well as HLAs.

Predictably, scRNA-seq combined with long-read RNA-seq can provide higher sensitivity and accurate full-length paired TCR sequences. Singh et al. (2019) combined targeted capture and long-read TCR and BCR mRNA transcription with short-read scRNA-seq to track the transcriptomic signature of expanded clonotypes from primary tumors and draining lymph nodes of breast cancer patients. Understanding gene regulation and function requires the ability to capture gene expression levels and isoform diversity at the single-cell level, in which short-read RNA-seq is limited in its capacity. Using Oxford Nanopore MinION sequencer to analyze individual murine B1a cells, Byrne et al. (2017) analyzed and identified different uses of complex isoforms in over a hundred genes, including surface receptors that determine B cell identity-determining surface receptors (e.g., CD19, CD20, and IGH). Multiple studies to date have shown that certain TCR clonotypes were expanded in the PBMCs or tissues of patients with autoimmune diseases. Still, the link between these TCRs and their functional relevance in the disease onset and development has not been identified, which requires refined studies of the gene transcriptome and the isoforms of TCR-expressing T cells. Thus, although there is no current application of long-read RNA-seq in autoimmune diseases, its future help in identifying complex etiologies can be foreseen.

Another promising application of long-read RNA-seq is in the field of HLAs. HLAs are a group of related proteins encoded by the MHC gene on human chromosome 6 and plays an essential role in autoimmune diseases. Previous *in silico* studies in our lab have shown that peptides with similar amino acid patterns may be presented to the same HLA due to structural similarities, thus initiating the autoimmune cascade (Gupta et al., 2022). Even though several analysis tools were developed to perform HLA typing from short RNA-seq reads using whole transcriptome data (Boegel et al., 2012; Kim and Pourmand, 2013; Buchkovich et al., 2017; Orenbuch et al., 2020; Chelysheva et al., 2021; Johansson et al., 2021), the large HLA genes (more than 5 kb) and the high degree of polymorphism within the class I (HLA-A, -B, and -C) and class II HLA (HLA-DR, -DQ, and -DP) often leads to ambiguous results in allele assignment. To this end, Cornaby et al. (2022) used long-read long sequencing of UMI-based high-resolution HLA typing and transcript quantification with a 99.68% overall HLA typing accuracy. Determining the profile of autoimmune-associated T cells requires deciphering the TCR and the HLA linkage. Thus, the introduction of long-read RNA-seq with the currently available scRNA-seq technology should allow a more in-depth study of innate, humoral, and T cell-mediated immunity in the future and will help provide a roadmap linking the pathogenesis of autoimmune diseases to the host immune response.

## 3 Structure study based on RNA sequencing results in TCR

### 3.1 Analysis of TCR-seq data

**FIGURE 3:**
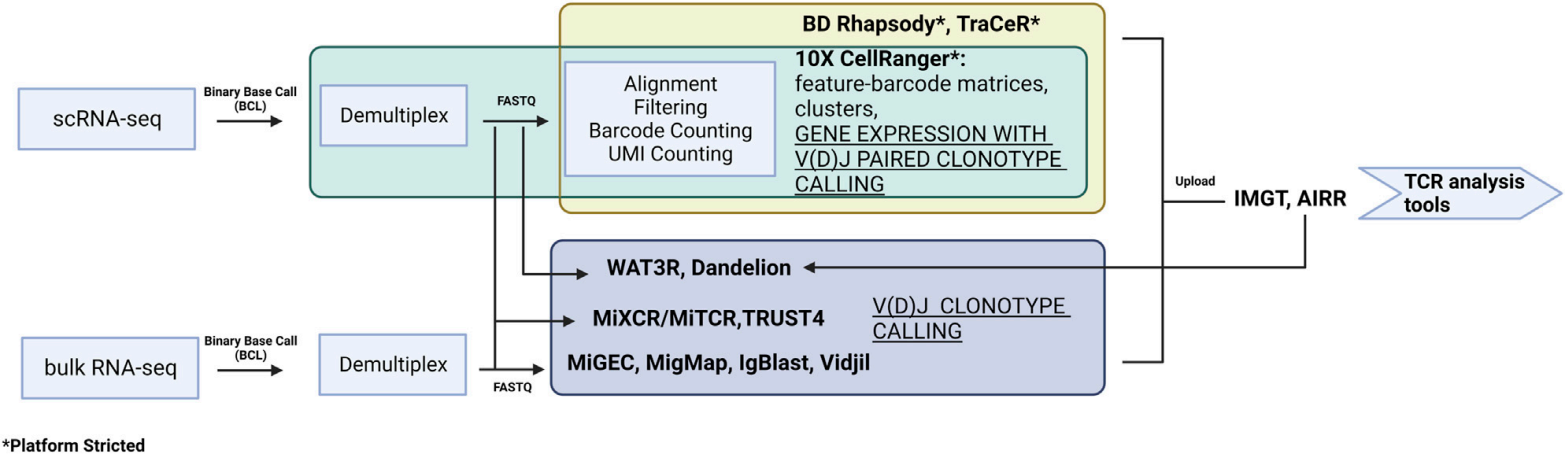
A summary of the pre-processing software for data from both scRNA-seq and bulk RNA-seq. The preprocessing relates to experimental design and library construction procedure and therefore can only follow a specific pipeline.

Retrieval of transcriptomic data enables the interrogation of multiple parameters simultaneously. More importantly, it allows for the examination of a targeted objective, e.g., the expression of novel TCR repertories in specific T cell subsets that are clinically detrimental in an autoimmune disease. Recent technology and platforms enable users to follow the analytical pipelines to generate meaningful results from transcriptomics to predictive structural modeling. Raw data needs to be pre-processed before it can be applied to the downstream TCR analysis (Figure 3). Depending on the platform used [e.g., 10X CellRanger for 10X Genomics, BD Rhapsody, TraCeR (Stubbington et al., 2016) for Fluidigm C1], the raw datasets are processed slightly differently but all generate expression matrix with TCR output files. There are also tools that specialize in extracting only repertoire information from FASTQ files. For example, MiXCR/MiTCR (Bolotin et al., 2015) and TRUST4 (Song et al., 2021) can process data from bulk RNA-seq and scRNA-seq data with and without V(D)J enrichment. MiGEC (Shugay et al., 2014), MigMap (Shugay and Davenport, 2018), IgBlast (Ye et al., 2013), and Vidjil (Giraud et al., 2014) can only work on bulk RNA-seq. Dandelion (Suo et al., 2023) is designed to work with Adaptive Immune Receptor Repertoire (AIRR) (Rubelt et al., 2017) -formatted input or 10X CellRanger VDJ output. WAT3R (Ainciburu et al., 2022) can process on 3′ single-cell RNA-seq data without V(D)J enrichment. Due to the high cost of library preparation and sequencing, there are also public databases containing V(D)J sequence information available for use, such as international ImMunoGeneTics information system (IMGT) (Lefranc et al., 2015) and AIRR. There is growing number of bioinformatic tools for TCR analysis. The output formats from pre-processing are different and the available downstream software varies, but most TCR analysis tools can recognize multiple formats.

Scirpy (Sturm et al., 2020) is a Python package that is an extension of Scanpy, which enables the user to visualize single-cell immune libraries and integrate them with transcriptomic data to characterize the TCR of single T cells. Scirpy supports multiple data formats, including 10X CellRanger, BD Rhapsody, TraCeR, Dandelion, or AIRR-compatible data. Scirpy enables the study of TCR chain configurations and explores clonotypes’ abundance, diversity, expansion, and overlap across samples, patients, or cell clusters. This software also allows analysis of CDR3 sequence length and the distribution of V(D)J gene usage. Specifically, Scirpy implements a sequence-alignment-based network that enables the clustering of cells into clonotypes based on having identical/similar CDR3 amino acid sequences, which offers the opportunities to identify cells that might recognize the same antigens.

Immunarch (Samokhina et al., 2022) is an R package which accepts all standard immuno-sequencing formats. It also automatically detects and parses uploaded data in formats including ImmunoSEQ, IMGT, MiXCR/MiTCR, MiGEC, MigMap, VDJtools, AIRR, and 10X CellRanger. Immunarch can annotate clonotypes using an external immune receptor database. The exclusive features include basic statistics such as CDR3 length distribution and clonotype abundance-more specifically, it can calculate the distribution of clonotypes per CDR3 length or clonotype spectratype. It can complete the analysis of repertoires dynamics, diversity, clonality, and overlap as well as compute V/J gene usage, and the distributions of kmers and sequence profiles.

The Loupe V(D)J Browser [10x Genomics Loupe V(D)J Browser 3.0.0] is a desktop application for Windows and macOS that allows users to analyze, search, and visualize V(D)J sequences and clonotypes. The Loupe V(D)J Browser identifies a sample’s most common paired αβ TCR chains. It filters clonotypes based on their antigen specificity or UMI number per antigen andexamines full-length V, D, and J amino acid and nucleotide sequences to detect variants in V(D)J transcripts, motifs within CDR3 regions, and compares clonotype frequencies between samples. It can be integrated with the Loupe Browser (formerly Loupe Cell Browser) to analyze data from different 10X genomics solutions. However, this tool has drawbacks since it is specifically designed to analyze 10X Genomics Single Cell Immune Profiling dataset.

ImmunoSEQ Analyzer (Adaptive Biotechnologies ImmunoSEQ Analyzer 3.0) is an online web-based tool for data exploration. Since the platform was developed only for ImmunoSEQ, it directly identifies V, D, and J genes and whole nucleotide sequences; non-productive sequences can be filtered out, and specific data values for immune sequencings, such as clonality, can be precomputed and visualized directly on the dashboard. Like Loupe V(D)J Browser, it provides basic statistics of clonotypes. In addition, the analyzer has tools for performing additional statistical tests and metrics on immune sequencing data. These include tools for clonotype diversity and tracking among samples. The main advantage of using this analyzer is that it contains an extensive database of TCR sequences, integrating millions of public data sequences and control samples.

VDJtools (Shugay et al., 2015) is an open-source software framework for TCR analysis based on Java. It is mainly used for post-analysis of clonotypes containing VDJ junction output for the following platforms: MiXCR/MiTCR, MiGEC, IgBlast, IMGT, ImmunoSEQ, VDJdb, Vidjil, MiXCR, ImmunoSEQ, and 10X CellRanger. VDJtools enables visualization of basic and advanced immune repertoires by applying different methods and strategies, including basic segment and segment usage, repertoire overlap, diversity analysis, data joining and clonotype tracking, and repertoire clustering.

scRepertoire (Borcherding et al., 2020) is an R package compatible and integrated with the R packages Trex for deep-learning-based autoencoding of TCR, which supports 10X CellRanger, AIRR, WAT3R, and TRUST4. scRepertoire is designed to obtain filter contig output from the pipeline, assign clonotypes according to the two TCR chains, and analyze the dynamics of clonotypes. It can be used for clonotype visualization, analysis of unique clonotypes, or clonal space quantification. Further features include clonal proportion analysis, sample similarity measures (scatter comparison between two samples), and overlap analysis for two or more samples. A unique feature is that the output data can be integrated with transcriptomic data [using Seurat (Satija et al., 2015), SingleCellExperiment (Amezquita et al., 2020), or Monocle 3 (Trapnell et al., 2014)].

There are also interactive databases available with known TCR sequences and clonotypes that can identify shared clones in multiple samples and explore the specificity of the immune response. An example is VDJdb (Shugay et al., 2018), a TCR sequence database with known antigenic specificity. The main goal of VDJdb is to facilitate access to information on the antigenic specificity of existing TCRs, i.e., the ability to identify certain epitopes in a specific MHC context. This database, which has been collecting and managing publicly available sequencing data obtained from TCRs with well-defined antigenic specificity, as well as data voluntarily shared by researchers, has been extended to a web interface that allows bulk querying of the AIRR dataset and identification of TCR sequence motifs associated with specific epitopes. There is also tcrdist3 (Mayer-Blackwell et al., 2021), an open-source python package based on distance-based TCR repertoire analysis capable of performing extensive TCR sequence analysis, including diversity analysis. The software utilizes meta-cloning concepts to group TCRs, i.e., a set of TCRs that are biochemically similar and likely to recognize the same antigen. The package has extended this to include support for gamma-delta TCRs.

Given these innovative tools, the challenges persist. Whether it is a pre-processing platform or a TCR analysis software, it is difficult for users to reach a uniform standard due to the many options, especially since most platforms can perform the same functions. Generally, the pre-processing relates to experimental design and library construction procedure following a specific pipeline (i.e., single-cell or bulk, sequencing platform, UMI integration). Therefore, it is imperative to develop a standard or “universal” pipeline that could support and simplify the process. Furthermore, most of the software is programming-based, which makes it necessary for users to have basic programming skills to operate and manipulate. A few available web or application-based platforms, which can meet the basic research needs, limit the ability to customize, and are not open-sourced or strictly product-based. Hence, these challenges are some of the major impediments that may discourage researchers interested in applying these tools for their research.

### 3.2 3D structural modeling

There is an array of TCR modeling platforms and capabilities including, but not limited to: Structural T Cell Receptor Modelling Tool (STCRPred) (Leem et al., 2018; Wong et al., 2019; Wong et al., 2020), TCRModel (Gowthaman and Pierce, 2018), and NetTCR (Montemurro et al., 2021) (Table 4). The former is a platform connected to SAbPred (Dunbar et al., 2016), initially constructed for 3D modeling and optimization of the B cell receptor (BCR), which also provides many of the same capabilities through SCALOP-TCR and TCRBuilder. SCALOP (Sequence-based Prediction of TCR CDR Canonical Form)-TCR is a sequence-based canonical form predictor for five of the six complementarity-determining regions (B1, B2, A1, A2, and A3) on a TCR. This provides an essential framework loop structure omitting side chains, compared to TCRBuilder, which may be more practical and include those interactions. TCRModel uses two modes: TCR-pMHC complex modeling (further discussed below) and unbound TCR modeling. The latter allows a simple model of the TCR, complete with any mutations, or by simply inputting the CDR3 sequences into the germline genes. Rosati et al. (2022) recently utilized this technology to model Crohn-associated invariant T (CAIT) cells with the paired TCR chain, which had been identified as an NKT type II population in Crohn’s Disease patients. NetTCR is a very limited platform. However, it may be helpful if the following criteria are met: known CDR3 sequence, satisfied with the provided three peptide sequences, and MHC-1 prediction will be exclusively for HLA-A*02:01 (Reynisson et al., 2020); while not strictly within the scope of TCR sequencing, MHC modeling can predict peptides to be presented to the TCR. This may be a useful tool within autoimmunity if the HLA is well known, as it is in diabetes. Notably, there also exist customized programs; for example, Jokinen et al. (2021) created TCRGP with which they were able to identify an exhausted, low functional T cell cluster that was enriched with Hepatitis B virus-targeting clonotypes, which they theorized could be pathogenic in causing hepatocellular carcinoma. Likewise, pipelines like this may be helpful in autoimmune disorders, especially those with a proposed viral or bacterial etiology.

**TABLE 4:** TCR (-pMHC) modeling platforms and capabilities.

Platform	TCRα	TCRβ	CDR3	MHC present	Output	Limitations
TCRModel	Yes	Yes	Yes	Yes	Unbound TCR, as well as TCR-pMHC complex modeling	NA
NetTCR	No	No	Yes	HLA-A*02:01 Only	List of predicted epitope binding	Only select from three peptide sequences
SCALOP-TCR	Yes	Yes	Yes	No	Predicts the structure of five CDRs (B1-2 and A1-3)	Does not include side chains
TCRBuilder	Yes	Yes	Yes	No	Multiple predicted conformations and an ensemble conformation would be returned	NA
TCRex	No	Yes	Yes	No	List of reactive epitopes	Only select from 93 viral and 5 cancer epitopes
TCRpMHCmodels	Yes	Yes	Yes	Class I only	TCR-pMHC complex modeling	Class I Only

### 3.3 Epitope prediction

**FIGURE 4:**
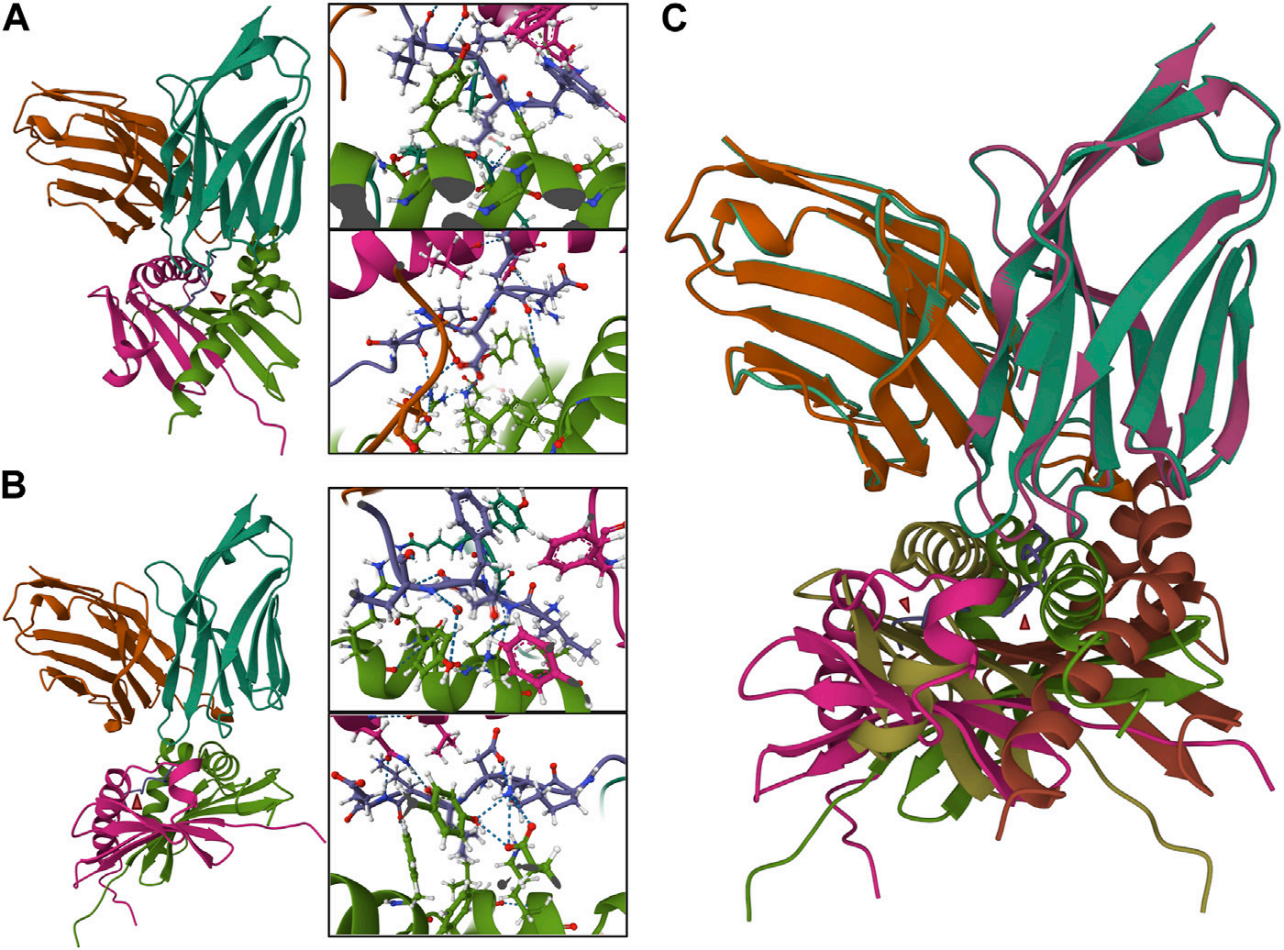
TCR-pMHC model containing autoantigens presented by SjD-susceptible HLA to the TCRs of selected patients. Two different peptides were selected to be presented by a SjD-susceptible HLA (DRA*01/DRB1*0301) to a selected paired TCR from SjD patient (VA 12-2, JA 13, CDR3A: CAVRIGGYQKVTF; VB 3-1, JB 2-3, CDR3B: CASSQEGREGRNTQYF). In molecular docking, a nine amino acid peptide is predicted to bind. The intermolecular contacts of this peptide are presented in the corresponding predictive model diagrams (aa1–4, upper, in order to clearly present the side chain amino acid order from right to left; aa5–9, lower, order from left to right). **(A)** NPWLILSEDRRQVRL, “WLILSEDRR” is predicted to bind. **(B)** FTFIQFKKDLKESMK, “IQFKKDLKE” is predicted to bind. **(C)** Superposition of the two TCR-pMHC models **(A,B)** showed a divergent presentation pattern. In **(A,B)**, Pink and green: HLA-DRA*01/DRB1*0301. Turquoise and orange: TCRα and ß chains. Purple: peptide.

Several programs have been written to predict what TCR will react against a given antigen. Programs predicting how epitopes dock in a TCR are limited but growing significantly recently (Table 4). The aforementioned TCRex is a platform that allows for selection from 93 viral and five cancer epitopes (Gielis et al., 2019). This platform enables users to train their custom model with machine learning, which is dependent on a manually curated catalog of pathology-associated TCR sequences (McPAS-TCR) (Tickotsky et al., 2017), VDJ database (VDJdb) (Shugay et al., 2018), and the ImmuneCODE-database (Nolan et al., 2020). For this platform and those to follow, splitting known autoantigens into shorter peptides and artificially docking those peptides may be the most useful. However, if the approach is to understand the etiology of the pathogenesis of the disease and potential triggers, viral/bacterial epitope mapping may also be useful. In this case nucleotide Basic Local Alignment Search Tool (BLASTn) may be a useful tool (Ladunga, 2002). TCRpMHCmodels is a tool for 3D modeling TCRs bound to peptides presented by a MCH class I (Jensen et al., 2019). Likewise, TCRmodel’s TCR-pMHC complex modeling is a very useful tool to either look at the interaction with a user-supplied peptide docked on a chosen MHC (either Class I or II) for both humans and mice (Gowthaman and Pierce, 2018). Our group has used COOT and PHENIX to predict pathogenic autoantigens presented by SjD-susceptible HLA, which has previously relied on superimposing chains on the crystal structure of solved peptide/HLA complexes on a LINUX system (Gupta et al., 2022). Now with TCRmodel we could further analyze the TCR-pMHC complex of autoantigens presented by SjD-susceptible HLA to selected patient’s TCR. With a web-based platform, this allows us to predict intermolecular contacts between peptide and HLA and cognate interactions between the TCR and peptide/HLA complex (Figure 4). While this technology has yet to be widely utilized in autoimmunity, Kasmani et al. (2023) used this program to show that CD8^+^ TCR avidity correlates with an exhausted fate during persistent infection by lymphocytic choriomeningitis virus in mice, where TCR sequences were paired with the peptide KAVYNFATC and the mouse class I MHC H-2D^b^.

Artificial intelligence (AI) has recently gained traction within the scientific community, and the epitope mapping field is no exception. Within the last 2 years, four new programs have been created: DECODE, TITAN, DeepTCR, and pMTnet. It should be noted that all of these programs utilize known biochemical reactivities (e.g., an amino acid present at specific residues as well as their interactions with the TCR and MHC/HLA). DECODE (DEcoding t Cell receptOr binDing rulEs) is a machine learning, customizable program that can allow users to select for specific reactivities (e.g., an amino acid at a particular residue) to further specify and customize the dataset for the end user (Papadopoulou et al., 2022). TITAN (Tcr epITope bimodal Attention Networks) is a bimodal neural network that explicitly encodes both TCR sequences and epitopes, which, interestingly, was able to identify previously unseen TCRs (Weber et al., 2021). The remaining two are more based on deep learning. DeepTCR analysis provides noise-depleted scRNA-Seq and *ex vivo* T cell assay results, which enables the user to identify rare subsets of TCRs and novel epitopes (Sidhom et al., 2022). And pMTnet (pMHC-TCR binding prediction network) was built to predict TCR-binding to neoantigens in human tumor genomics datasets. Notably, this program only utilized the CDR3β sequence of the TCR, epitope sequence, and class I MHC allele (Lu et al., 2021). While these technologies have been restricted to oncogenic research, AI is becoming more available both within research environments and from private companies. Utilization of this technology may lead to the identification of novel pathogenic T cells with specific TCRs or novel autoantigens driving autoimmune disease pathology.

## 4 Discussion

The rapid advances in RNA-seq technology have enabled the analysis of the transcriptome in various ways, both serving to further the understanding of genome function and crucially for studying mRNA splicing and rearrangements. Many alternative sequencing platforms are currently available, and short-read RNA-seq combined with single-cell technology is currently the mainstay. However, the future of autoimmune disease research lies in efficient long-read RNA-seq. The sequence and rearrangement of TCR are closely related to the pathogenesis of autoimmune diseases, and HLA genes are well-documented genetic risk factors for the development of certain autoimmune diseases. While current studies focus on HLA typing, the clonal expansion of the immune repertoire, or CDR3 motifs in patients (differentiating from healthy individuals), in the future, by sequencing individual T cells, we will not only be able to obtain the sequence of TCRs, but we will also be able to obtain transcriptomic data of T cells expressing TCRs, from which we can analyze the subtypes of cells. Combined with accurate HLA typing and artificial intelligence (AI)-based structural analysis, we can predict autoimmune TCR-pMHC complexes even before the onset of the disease. Identifying the autoantigen and TCR repertoire and generating a predictive autoimmune response will have a significant potential for clinical applications and also advances our knowledge of autoimmune diseases. More importantly, the approach will bring tremendous potential in infectious diseases, from which we can optimize vaccine development to target individual antigen-specific TCR enhancements. The main issues currently hindering the adoption of long-read sequencing are the increased cost per base and the higher error rate compared to short-read sequencing. Unlike short-read sequencing where errors are usually clustered at both ends of the read, long-read sequencing errors are random and can be effectively corrected by multiple sequencing events. Still, these issues will gradually be overcome as technology advances. With the vigorous development of the RNA field, multidisciplinary research can bring breakthroughs in studying autoimmune diseases.
